# Supplementary material for: Speckle modulation enables high-resolution wide-field human brain tumor margin detection and in vivo murine neuroimaging
Source: Sci Rep. 2019 Jul 17;9:10388. doi: 10.1038/s41598-019-45902-9 (PMC6637128; doi:10.1038/s41598-019-45902-9)
Supplement: Supplementary file 1 — Supplementary Info File #1 [file 41598_2019_45902_MOESM1_ESM.pdf]

Supplementary Information

Speckle modulation enables high-resolution wide-field human brain tumor margin detection and *in vivo* murine neuroimaging

Derek Yecies[1,2] ǂ, Orly Liba[1,3,4,5] ǂ, Elliott D. SoRelle[1,4,5,6], Rebecca Dutta[1,4], Edwin Yuan[1,4,7], Hannes Vogel[8], Gerald A. Grant[2], & Adam de la Zerda[1,3,4,5,6,9]*

[1]Stanford University Department of Structural Biology, [2] Stanford University Department of Neurosurgery, [3]Stanford University Department of Electrical Engineering, [4]Molecular Imaging Program at Stanford, [5]Bio-X Program at Stanford, [6]Biophysics Program at Stanford University, [7]Applied Physics Program at Stanford University, [8]Stanford Pathology, [9]The Chan Zuckerberg Biohub, San Francisco, CA 94158

ǂ Equal contribution
* Corresponding author

Supplementary Figures

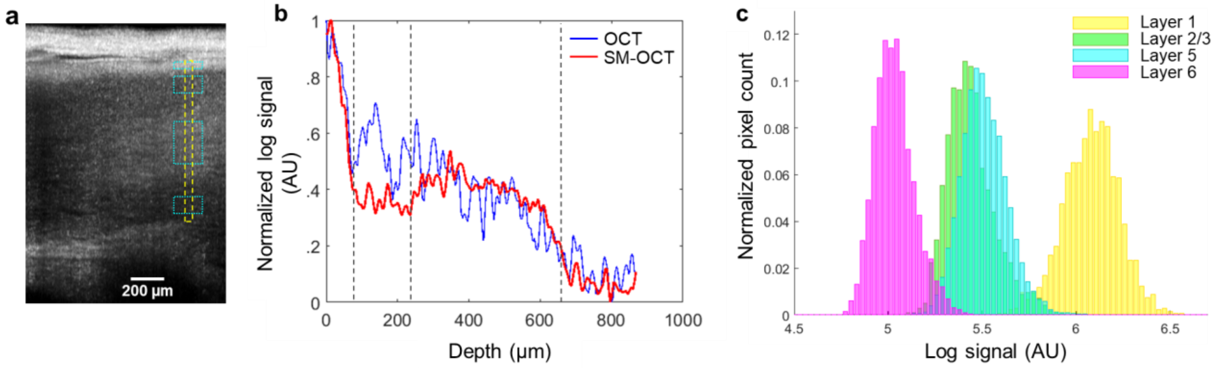

Supplementary Figure 1: SM-OCT imaging of mouse brain *in vivo* reveals cortical layers.

a) SM-OCT B-scan of mouse cortex, showing the areas used for quantifying the signal in the following analysis. b) the signal intensity of OCT and SM-OCT as a function of depth. The region of the signal is shown as a yellow dashed line in (a). c) Normalized histograms of the SM-OCT signal intensity, on the regions shown by cyan rectangles in (a).

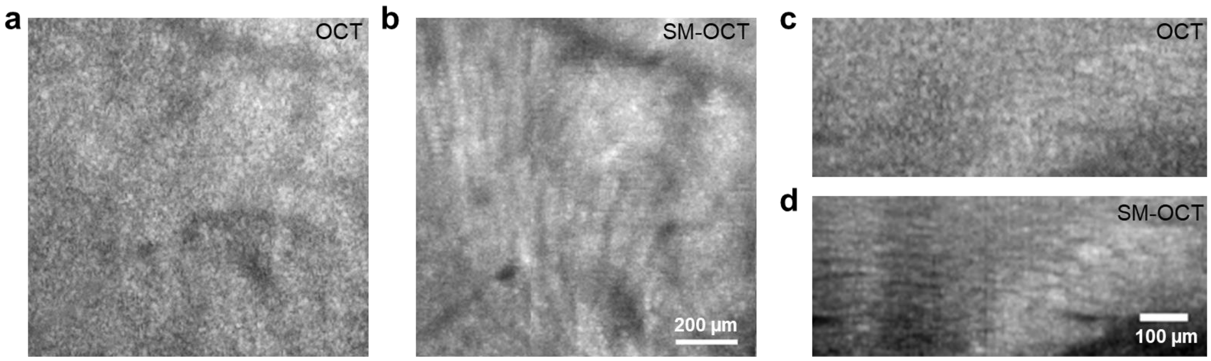

Supplementary Figure 2: Comparison between SM-OCT and OCT images of mouse white-matter structures *in vivo*.

a, b) OCT and SM-OCT (respectively) axial close up views (*en face*) of the cingulum bundle. The structures of the fascicles are revealed through speckle removal. c, d) OCT and SM-OCT (respectively) coronal close up views (B-scans) of the white matter structures, revealed in high-resolution when using SM-OCT.

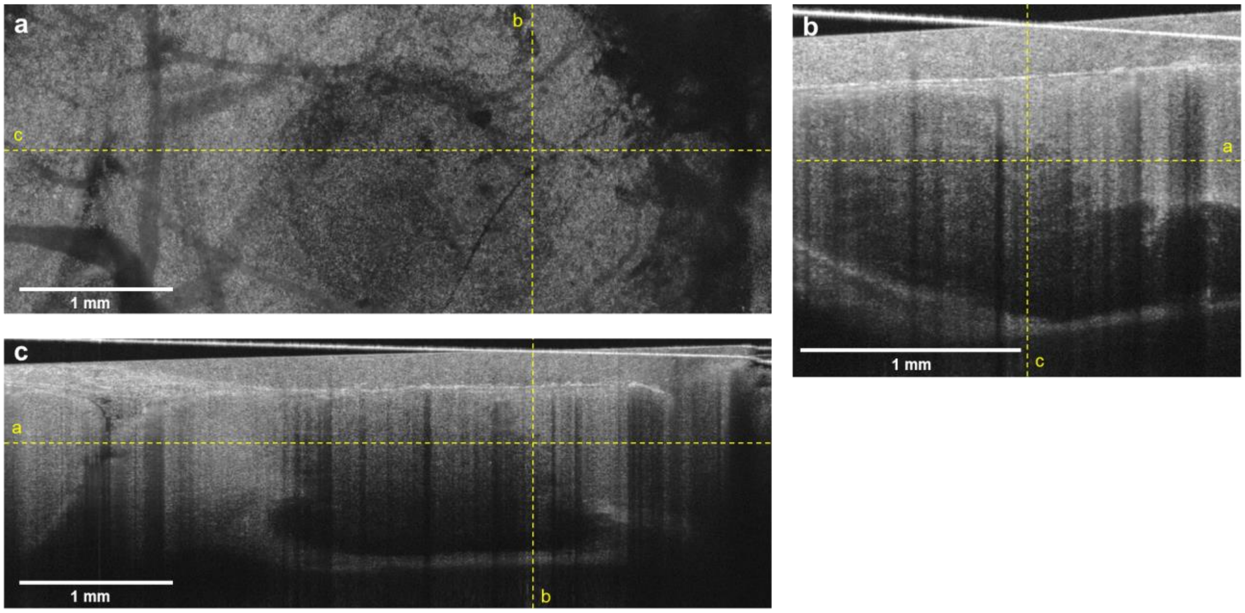

Supplementary Figure 3: OCT volume, corresponding to the SM-OCT volume in Fig. 3.
Axial (a), sagittal (b) and coronal (c) views of the GBM tumor margin, imaged with OCT *in vivo*. Compared to the SM-OCT volume, the speckle noise in OCT obscures the fine structures at the tumor margin.

Supplementary movie captions

Supplementary Movie 1: OCT and SM-OCT comparison, coronal view of mouse brain *in vivo*

Supplementary Movie 2: SM-OCT, axial view of mouse brain *in vivo*

Supplementary Movie 3: OCT and SM-OCT comparison, axial view of mouse brain *in vivo* with close-up views

Supplementary Movie 4: Enhanced SM-OCT, axial view of mouse brain *in vivo*

Supplementary Movie 5: SM-OCT imaging of mouse brain with tumor, axial view

Supplementary Movie 6: SM-OCT imaging of mouse brain with tumor, coronal view

Supplementary Movie 7: SM-OCT imaging of mouse brain with tumor, sagittal view
